# Rheumatoid Arthritis Associated with Dry Eye Disease and Corneal Surface Damage: A Nationwide Matched Cohort Study

**DOI:** 10.3390/ijerph20021584

**Published:** 2023-01-15

**Authors:** Shih-Chung Lai, Chien-Wun Wang, Yu-Ming Wu, Ying-Xiu Dai, Tzeng-Ji Chen, Hsiang-Ling Wu, Yih-Giun Cherng, Ying-Hsuan Tai

**Affiliations:** 1 Department of Ophthalmology, Shuang Ho Hospital, Taipei Medical University, New Taipei City 23561, Taiwan; 2 Department of Ophthalmology, School of Medicine, College of Medicine, Taipei Medical University, Taipei 11031, Taiwan; 3 Department of Anesthesiology, Shuang Ho Hospital, Taipei Medical University, New Taipei City 23561, Taiwan; 4 Department of Anesthesiology, School of Medicine, College of Medicine, Taipei Medical University, Taipei 11031, Taiwan; 5 Department of Dermatology, Taipei Veterans General Hospital, Taipei 11217, Taiwan; 6 School of Medicine, National Yang Ming Chiao Tung University, Taipei 11221, Taiwan; 7 Department of Family Medicine, Taipei Veterans General Hospital, Taipei 11217, Taiwan; 8 Department of Family Medicine, Taipei Veterans General Hospital, Hsinchu Branch, Hsinchu 31064, Taiwan; 9 Department of Anesthesiology, Taipei Veterans General Hospital, Taipei 11217, Taiwan

**Keywords:** autoimmune disease, corneal erosion, keratoconjunctivitis sicca, peripheral ulcerative keratitis, risk factor

## Abstract

Rheumatoid arthritis is potentially connected to ocular disorders, such as corneal inflammation and lacrimal gland destruction. This study aimed to evaluate the risk of dry eye disease (DED) and corneal surface damage among patients with rheumatoid arthritis. In a nationwide cohort study, we utilized Taiwan’s National Health Insurance research database and conducted propensity score matching to compare the risks of DED and corneal surface damage between patients with and without rheumatoid arthritis. Proportional hazards regression analyses were used to calculate the adjusted hazard ratio (aHR) and 95% confidence interval (CI) for the outcomes of interest. The matching procedure generated 33,398 matched pairs with 501,377 person-years of follow-up for analyses. The incidence of DED was 23.14 and 10.25 per 1000 person-years in patients with and without rheumatoid arthritis, respectively. After adjusting for covariates, rheumatoid arthritis was significantly associated with DED (aHR: 2.03, 95% CI: 1.93–2.13, *p* < 0.0001). The association was generally consistent across the subgroups of age, sex, use of systemic corticosteroids, and different comorbidity levels. In addition, patients with rheumatoid arthritis had a higher risk of corneal surface damage (aHR: 1.36, 95% CI: 1.21–1.51, *p* < 0.0001) compared to control subjects. Other independent factors for corneal surface damage were age and sleeping disorders. Rheumatoid arthritis was associated with an increased risk of DED and corneal surface damage. Ophthalmological surveillance is required to prevent vision-threatening complications in this susceptible population.

## 1. Introduction

Dry eye disease (DED) is a growing epidemic worldwide, with an estimated prevalence of 5% to 64% [1,2]. In Taiwan, epidemiological studies have reported a prevalence rate of DED ranging from 4.6% to 33.7% [3,4]. DED is potentially associated with ocular surface injury and visual disturbance, which makes it the most common diagnosis in the eye care service [5]. Additionally, DED adversely affects the quality of life and decreases the workforce productivity, placing a large financial burden on patients and medical care system [6,7].

The pathogenesis of DED is multifaceted and can be classified into aqueous-deficient and evaporative subtypes [8]. An unstable tear film, hyperosmolarity, inflammation and damage of ocular surface, and neurosensory abnormalities are deemed as the major contributing factors of DED [9]. DED might develop into superficial punctate keratitis, filamentary keratitis, and superior limbic keratoconjunctivitis [10]. Importantly, disruption of the corneal barrier function might result in serious corneal surface pathologies, such as corneal erosion, ulcers, and opacity [10].

Rheumatoid arthritis (RA) is one of the most common systemic autoimmune diseases, with an increasing prevalence rate by 7.4% from 1990 to 2017 [11]. In Taiwan, the annual incidence rate of RA was 15.8 cases per 100,000 population, and the prevalence rate increased steadily from 57.7 in 2000 to 99.6 per 100,000 people in 2007 [12,13]. RA is characterized by progressive articular damage, potentially leading to functional disability and premature mortality [14]. RA is also associated with extra-articular manifestations, potentially involving the eyes. Common ocular manifestations of RA include corneal inflammation, lacrimal gland dysfunction, and uveitis, sharing similar pathogenic mechanisms to DED [15]. Noticeably, studies have shown that DED is more likely to progress into corneal erosion and ulcers among patients with systemic autoimmune diseases [15,16].

The relationship between RA and DED or corneal surface damages is still not fully clarified due to some methodological limitations of previous studies, including a small sample size (*n* < 1000) [17,18,19,20,21,22,23], insufficient adjustment for confounders [17,18,19,20,22,23], and restriction to specific medical institutions [17,18,19,21,22,23] or populations [19,20]. Furthermore, relevant risk factors for corneal surface damage among patients with RA remain largely unknown. Accordingly, we conducted a population-based cohort study using Taiwan’s National Health Insurance (NHI) research database to clarify the temporal association between RA and aqueous-deficient DED or corneal surface damage. The objective of this study was to compare the risks of DED and corneal surface injury between patients with and without RA. In addition, we also sought to identify independent factors for corneal diseases associated with RA. Based on the current evidence [17,18,19,20,21,22,23], we hypothesized that RA was associated with more aqueous-deficient DED and corneal surface damage in the nationwide autoimmune population.

## 2. Material and Methods

### 2.1. Source of Data

This study was approved by the Institutional Review Board of Taipei Medical University (approval no. TMU-JIRB-N202210011; date of approval on 6 October 2022). Written informed consent was waived by the Institutional Review Board due to the retrospective nature of this study. All methods of this study were performed in accordance with the standards of the Helsinki Declaration-2013 and relevant study guidelines [24]. Taiwan’s National Health Insurance program was launched in March 1995 and covers more than 99% of Taiwanese and non-Taiwanese working or studying in Taiwan. The NHI research database contains comprehensive registration files and original claims data of the insured beneficiaries, including demographic attributes, medical diagnoses, and prescription drugs. Research articles based on the NHI research database have been published in prominent scientific journals [25,26,27]. The study subjects were included from the three Longitudinal Health Insurance Databases (LHID2000, LHID2005, and LHID2010), which contains original claims data of one million randomly sampled subjects from the original NHI research database in the years 2000, 2005, and 2010, respectively [28].

### 2.2. Inclusion and Exclusion Criteria

Inclusion criteria were patients who had at least two rheumatology clinic visits with the diagnosis of RA from 1 January 2002 to 30 June 2013. The International Classification of Diseases, 9th Revision, Clinical Modification (ICD-9-CM) codes were used to identify the diagnoses of RA, coexisting diseases, and ocular diseases (Supplementary Table S1). The index date was defined as the date of the first RA diagnosis. We excluded patients who had any diagnosis of DED, corneal ulcers, recurrent corneal erosion, corneal opacity, interstitial and deep keratitis, corneal neovascularization, ocular burns, or open globe injury in the ophthalmology service before the index date. We also excluded patients who had been prescribed eye lubricants before the index date. Patients who died during the follow-up period were also excluded.

### 2.3. Study Outcome

The primary outcome was DED, which was defined as the diagnosis made twice by certified ophthalmologists with prescriptions of cyclosporine ophthalmic emulsion (Restasis^®^) treatment in the ophthalmology service (Supplementary Table S1). In the reimbursement regulations of Taiwan’s National Health Insurance, Restasis^®^ ophthalmic emulsion is indicated when patients have a Schirmer test score of <5 mm in 5 min [4,29]. The secondary outcomes were secondary Sjögren’s syndrome (SS) and severe corneal surface damages, which were defined as any diagnosis of corneal ulcers, recurrent corneal erosion, or corneal opacity made twice by certified ophthalmologists.

### 2.4. Baseline Patient Characteristics

Monthly insurance premium was classified into 0–500, 501–800, and >800 USD. We used the ICD-9-CM codes of physicians’ diagnoses within 24 months before the index date to identify the following coexisting diseases, chosen on the basis of data availability and physiological plausibility: hypertension, diabetes mellitus, ischemic heart disease, chronic obstruction pulmonary disease, chronic liver disease, chronic kidney disease, cerebrovascular disease, thyroid disease, depressive disorder, anxiety disorder, sleeping disorder, and malignancy (Supplementary Table S1) [2]. The Charlson comorbidity index score was used to assess the comorbidity condition of studied subjects [30]. The concurrent use of systemic corticosteroids prescribed within 180 days after the index date was also examined in the analysis. We also evaluated the numbers of hospitalizations and emergency visits within 24 months before the index date to examine the level of medical resource utilization of the included subjects.

### 2.5. Statistical Analysis

Continuous variables were expressed as the mean ± standard deviation or median with interquartile range. Categorical variables were presented as the frequency and percentage. We used a non-parsimonious multivariable logistic regression model to obtain a propensity score for people with or without RA. Each RA patient was matched to a non-RA control using a greedy matching algorithm within a tolerance limit of 0.05 and without replacement to adjust for the distributions of age, sex, and monthly insurance premium between the two groups [31]. The balance of the baseline covariate distribution between matched pairs was evaluated using absolute standardized mean difference [32]. Multivariable proportional hazards regressions models were used to calculate the adjusted hazard ratio (aHR) and 95% confidence interval (CI) for the ophthalmological outcome. Factors controlled in the multivariable models were age, sex, monthly insurance premium, coexisting diseases, Charlson comorbidity index score, use of systemic corticosteroids, number of hospitalizations, and number of emergency room visits. We also used Kaplan–Meier curves and log-rank tests to compare the cumulative incidence of study outcomes between the two groups. Subgroup analyses were also conducted by age ≥ or <65 years, male or female, different Charlson comorbidity index scores, and use of systemic corticosteroids or not. A two-sided level of 0.05 was considered statistically significant. All the statistical analyses were conducted using Statistics Analysis System (SAS), Version 9.4 (SAS Institute Inc., Cary, NC, USA).

## 3. Results

### 3.1. Baseline Patient Characteristics

The propensity score matching analysis generated 33,398 matched pairs with 501,377 person-years of follow-up for comparison (Supplementary Figure S1). Table 1 shows the baseline patient and clinical characteristics of the RA and non-RA subjects. Of note, patients with RA were more likely to have more coexisting diseases, higher Charlson comorbidity index scores, use of systemic corticosteroids, and higher number of hospitalizations and emergence room visits.

### 3.2. Risk of Dry Eye Disease

**Figure 1:**
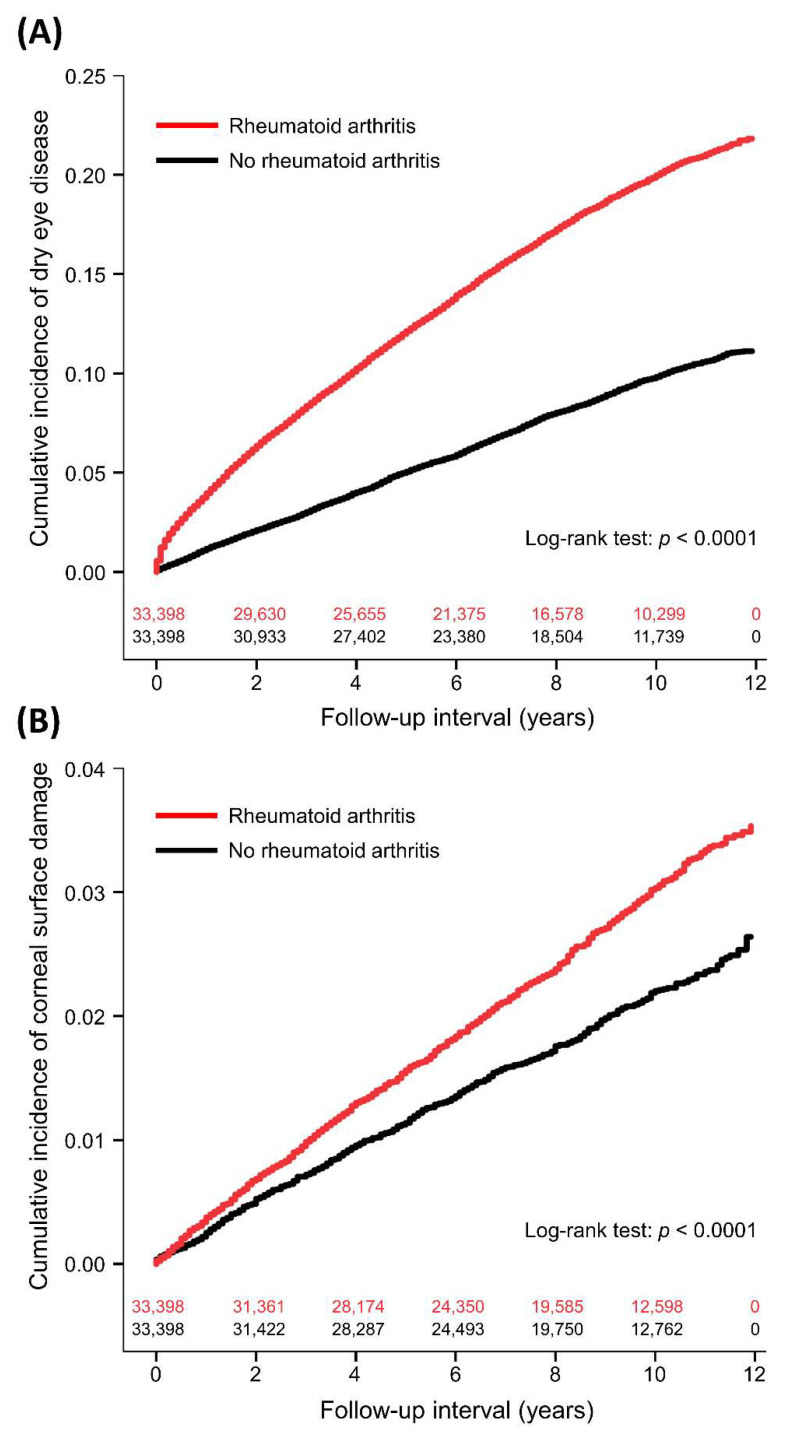
Cumulative incidence of dry eye disease (**A**) and corneal surface damage (**B**) between patients with and without rheumatoid arthritis with number of subjects at risk.

In the follow-up period, the incidence of DED was 23.14 and 10.25 per 1000 person-years in the RA and non-RA groups, respectively (Table 2). The duration between enrollment and DED diagnosis was a median of 3.2 (interquartile range: 1.2–6.0) years in the RA patients and 4.3 (1.9–6.9) years in the non-RA controls (*p* < 0.0001). Table 3 demonstrates the results of univariate and multivariable proportional hazards regression analyses for DED. RA was significantly associated with more DED compared to non-RA controls (aHR: 2.03, 95% CI: 1.93–2.13, *p* < 0.0001). The cumulative incidence of DED in the two groups is shown in Figure 1A. RA was also associated with increased SS (aHR: 4.11, 95% CI: 3.69–4.58, *p* < 0.0001). Other independent factors for DED were age (aHR: 1.02), sex (male vs. female, aHR: 0.46), monthly insurance premium (501–800 vs. 0–500 USD, aHR: 0.78; ≥801 vs. 0–500 USD, aHR: 1.02), hypertension (aHR: 0.90), chronic liver disease (aHR: 1.25), thyroid disease (aHR: 1.48), anxiety disorder (aHR: 1.27), sleeping disorder (aHR: 1.13), Charlson comorbidity index (1 vs. 0, aHR: 1.22; 2 vs. 0, aHR: 1.16; ≥3 vs. 0, aHR: 0.68), use of systemic corticosteroids (aHR: 1.36), and number of hospitalizations (1 vs. 0, aHR: 0.88; 2 vs. 0, aHR: 0.94; ≥3 vs. 0, aHR: 0.71). Subgroup analyses showed consistent associations between RA and DED, including different age groups, male or female, different Charlson comorbidity index scores, and use of systemic corticosteroids or not (Table 4).

### 3.3. Risk of Corneal Surface Damage

The incidence of corneal surface damage was 3.07 and 2.23 per 1000 person-years in the RA and non-RA groups, respectively (Table 2). The time to corneal surface damage was a median of 4.1 years (interquartile range: 1.8–7.1) in the RA patients and 4.0 years (interquartile range: 1.8–6.8) in the non-RA controls (*p* = 0.7746). After adjusting for covariates, RA was significantly associated with more corneal surface damage compared to non-RA controls (aHR: 1.36, 95% CI: 1.21–1.51, *p* < 0.0001; Table 5 and Figure 1B). Further analyses showed that RA was significantly associated with increased risks of corneal ulcer (aHR: 1.30, 95% CI: 1.11–1.52, *p* = 0.0009) and recurrent corneal erosion (aHR: 1.99, 95% CI: 1.55–2.55, *p* < 0.0001). Other independent factors for corneal surface damage were age (aHR: 1.01) and sleeping disorder (aHR: 0.80).

## 4. Discussion

In the present study, we found that RA was significantly associated with increased DED and corneal surface damages in a population-based matched cohort. The association was generally consistent across the subgroups of age, sex, use of systemic corticosteroids, and varying comorbidity levels. We also found some independent factors for corneal diseases associated with RA, providing evidence in early identification and prophylaxis for severe corneal morbidities. Our results highlight the need for regular ophthalmology follow-up for potential corneal complications among patients with RA.

Few studies have compared the incidence of DED and corneal diseases between RA and non-RA subjects. Two studies evaluated the association between RA activity and DED [17,21]. Three case reports demonstrated the complication of corneal ulcers in patients with RA [18,19,22]. Another study reported that 1.9% patients with RA had inflammatory ocular diseases in an older male population but did not analyze the relative risk [20]. DED is a highly prevalent disease [1,2,3,4], and there is little evidence indicating the risk difference in DED between RA and general population. Our results suggested that patients with RA had an increased risk of DED and corneal surface damages, filling the knowledge gap in the field of eye care for RA. A recent meta-analysis reported that DED is the most common ocular manifestation of RA with an estimated prevalence of 16%, which is slightly lower than our results [33]. Additionally, our study identified several risk factors for DED and corneal surface damages associated with RA, which were not reported in previous studies [17,18,19,20,21,22,23]. These findings might be useful in the risk stratification and early prevention for serious ocular complications.

The biology and pathogenesis of DED and peripheral ulcerative keratitis in RA are still not clearly elucidated. Current evidence suggests that lymphocyte and plasma cell infiltration in cornea and lacrimal gland destruction are possibly responsible for RA-associated DED [34]. In addition, destructive inflammatory cells in the marginal corneal cause progressive stromal degradation and thinning, corneal perforation, and ulceration [15,16]. Both T cells and antibodies associated with RA are involved in the corneal inflammation [35]. Furthermore, cornea-resident antigen-presenting cells, proinflammatory cytokines and chemokines, and the imbalance between matrix metalloproteinases and tissue inhibitors also contribute to corneal epithelial destruction and disease progression in RA [18,36,37].

Our findings highlight the importance of regular ophthalmology examinations for potential corneal manifestations in patients with RA. Early recognition and treatment of DED in RA are essential in preventing progressive corneal injury and irreversible vision impairment [38]. In an in vivo confocal microscopy study, Villani et al. found that the dendritic cell density of the cornea and levels of interleukin-1α and interleukin-6 of tear fluid were significantly reduced by systemic prednisone and methotrexate in RA patients with secondary SS, but this finding was not observed in those without SS [37]. These results suggest that the immunopathogenesis of corneal surface inflammation in RA might be different between SS and non-SS patients [37]. It remains unclear whether standard systemic treatment for RA (e.g., corticosteroids, immunosuppressive agents, and biologic therapy) is effective in preventing the occurrence of DED or peripheral ulcerative keratitis [39]. Further studies are needed to clarify the molecular process and prophylactic measures of DED and related complications in RA with or without secondary SS.

Our study had several strengths to detect the putative relationship between RA and corneal pathologies. First, a large-scale nationwide cohort was used to increase the statistical power and generalizability of study results. The subjects were followed up for up to 12 years to evaluate the temporal association of RA with corneal diseases. Second, our analyses considered a comprehensive list of patient and clinical factors to better clarify the independent association between RA and ocular complications. However, there were some limitations to our study. Firstly, our data did not contain information about objective physical measures (e.g., ocular test results), biochemical profiles (e.g., autoimmunity and inflammation markers), and clinical data on RA management (e.g., types and doses of disease-modifying antirheumatic drugs and biologic agents) that were not available in the NHI research database. Secondly, the activity and severity of RA could not be evaluated because of data unavailability. Therefore, whether RA activity is linked to development and severity of DED or corneal damages remains unclear. However, our multivariable analyses showed that use of systemic corticosteroids after diagnosis of RA was linked to a higher risk of DED. This finding might partly reflect a positive association between RA severity and DED risk. Thirdly, the study cohort did not include patients with subclinical DED or corneal inflammation who did not seek conventional eye care service. Fourthly, the prescriptions of cyclosporine ophthalmic emulsion as a part of primary outcome ascertainment might have underestimated the incidence of DED in this study. Fifthly, we did not analyze the economic impact of RA-associated DED and corneal surface injury due to a lack of accurate data on the costs of related treatments in the NHI research database. Lastly, our cohort was only followed up until 31 December 2013, due to the regulations of the NHI research database.

## 5. Conclusions

Patients with RA had an increased risk of DED and corneal surface damages compared to non-RA controls in the 12 year population-based cohort. Our study identified several risk factors for corneal surface injury, providing an implication for early identification and prevention for serious corneal sequela in patients with RA. Regular ophthalmology surveillance might be needed to mitigate the adverse effect of corneal inflammation on this susceptible population. Future studies are warranted to clarify the biological mechanism and to evaluate the effective prophylactic and therapeutic strategies for corneal complications in RA.

## Figures and Tables

**Table 1:** Baseline characteristics of patients with and without rheumatoid arthritis.

	RA *n* = 33,398		Non-RA *n* = 33,398		ASMD
Age (years), mean (SD)	49.6	16.2	49.6	16.2	<0.0001
Sex, male, *n* (%)	11,306	33.9	11,306	33.9	<0.0001
Insurance premium (USD/month), *n* (%)					<0.0001
0–500	14,184	42.5	14,184	42.5	
501–800	10,868	32.5	10,868	32.5	
≥801	8346	25.0	8346	25.0	
Coexisting diseases, *n* (%)					
Hypertension	8136	24.4	5630	16.9	0.2551
Diabetes mellitus	3638	10.9	2392	7.2	0.2538
Ischemic heart disease	3271	9.8	1774	5.3	0.3641
Chronic obstructive pulmonary disease	2495	7.5	1481	4.4	0.3054
Chronic liver disease	3973	11.9	2149	6.4	0.3720
Chronic kidney disease	442	1.3	217	0.7	0.3960
Cerebrovascular disease	1583	4.7	1155	3.5	0.1812
Thyroid disease	834	2.5	367	1.1	0.4604
Depressive disorder	433	1.3	231	0.7	0.3498
Anxiety disorder	5084	15.2	2589	7.8	0.4186
Sleeping disorder	4915	14.7	2634	7.9	0.3864
Malignancy					
Charlson comorbidity index					0.1196
0	26,645	79.8	28,861	86.4	
1	5718	17.1	3368	10.1	
2	860	2.6	929	2.8	
≥3	175	0.5	240	0.7	
Use of systemic corticosteroids, *n* (%)	11,383	34.1	4589	13.7	0.6491
Number of hospitalizations, *n* (%)					0.1591
0	28,191	84.4	30,042	90.0	
1	3612	10.8	2452	7.3	
2	941	2.8	533	1.6	
≥3	654	2.0	371	1.1	
Number of ER visits, *n* (%)					0.2135
0	24,594	73.6	27,546	82.5	
1	5464	16.4	3978	11.9	
2	1857	5.6	1063	3.2	
≥3	1483	4.4	811	2.4	

Abbreviations: ER = emergency room; RA = rheumatoid arthritis; SD = standard deviation; ASMD = absolute standardized mean difference; USD = United States dollar.

**Table 2:** Risk of dry eye disease and corneal surface damage for patients with and without rheumatoid arthritis.

	RA *n* = 33,398		Non-RA *n* = 33,398		Outcome Risk	
Study Outcome	Incident Case	Incidence per 1000 Person-Years	Incident Case	Incidence per 1000 Person-Years	aHR (95% CI) ^†^	*p*
Dry eye disease	5594	23.14	2661	10.25	2.03 (1.93–2.13)	<0.0001
Sjögren’s syndrome	2072	7.95	425	1.57	4.11 (3.69–4.58)	<0.0001
Corneal surface damage	825	3.07	601	2.23	1.36 (1.21–1.51)	<0.0001
Corneal ulcer	404	1.51	308	1.14	1.30 (1.11–1.52)	0.0009
Recurrent corneal erosion	194	0.72	103	0.38	1.99 (1.55–2.55)	<0.0001
Corneal opacity	227	0.85	193	0.72	1.10 (0.90–1.35)	0.3517

Abbreviations: aHR = adjusted hazard ratio; CI = confidence interval; RA = rheumatoid arthritis. ^†^ Adjusted for age (continuous), sex, insurance premium (categorical), coexisting diseases, Charlson comorbidity index score, use of systemic corticosteroids, number of hospitalizations, and number of emergency room visits.

**Table 3:** Univariate and multivariable analyses for dry eye disease.

	Univariate			Multivariable		
	cHR	95% CI	*p*	aHR	95% CI	*p*
Rheumatoid arthritis	2.24	2.14–2.35	<0.0001	2.03	1.93–2.13	<0.0001
Age (years)	1.02	1.02–1.02	<0.0001	1.02	1.01–1.02	<0.0001
Sex, male vs. female	0.45	0.43–0.48	<0.0001	0.46	0.43–0.48	<0.0001
Insurance premium (USD/month)			<0.0001			<0.0001
501–800 vs. 0–500	0.74	0.70–0.78	<0.0001	0.78	0.74–0.82	<0.0001
≥801 vs. 0–500	0.74	0.70–0.78	<0.0001	1.02	0.96–1.09	0.5089
Coexisting diseases						
Hypertension	1.34	1.28–1.41	<0.0001	0.90	0.85–0.96	0.0008
Diabetes mellitus	1.32	1.23–1.41	<0.0001	0.93	0.86–1.00	0.0560
Ischemic heart disease	1.48	1.37–1.59	<0.0001	1.05	0.97–1.13	0.2524
COPD	1.24	1.13–1.34	<0.0001	0.96	0.88–1.04	0.3140
Chronic liver disease	1.47	1.38–1.57	<0.0001	1.25	1.17–1.34	<0.0001
Chronic kidney disease	1.29	1.05–1.59	0.0163	0.97	0.78–1.20	0.7752
Cerebrovascular disease	1.31	1.19–1.45	<0.0001	1.00	0.90–1.11	0.9979
Thyroid disease	2.11	1.87–2.39	<0.0001	1.48	1.31–1.67	<0.0001
Depressive disorder	1.59	1.32–1.92	<0.0001	1.08	0.89–1.31	0.4291
Anxiety disorder	1.74	1.64–1.84	<0.0001	1.27	1.19–1.36	<0.0001
Sleeping disorder	1.60	1.50–1.70	<0.0001	1.13	1.05–1.20	0.0005
Malignancy	1.39	1.24–1.57	<0.0001	1.11	0.98–1.25	0.0983
Charlson comorbidity index			<0.0001			<0.0001
1 vs. 0	1.58	1.50–1.67	<0.0001	1.22	1.15–1.29	<0.0001
2 vs. 0	1.31	1.16–1.47	<0.0001	1.16	1.03–1.32	0.0142
≥3 vs. 0	0.77	0.56–1.06	0.1047	0.68	0.50–0.94	0.0198
Use of systemic corticosteroids	1.72	1.64–1.80	<0.0001	1.36	1.30–1.43	<0.0001
Number of hospitalizations			0.0102			0.0003
1 vs. 0	1.05	0.97–1.13	0.2627	0.88	0.81–0.95	0.0012
2 vs. 0	1.26	1.10–1.45	0.0013	0.94	0.81–1.09	0.4278
≥3 vs. 0	1.01	0.84–1.22	0.9028	0.71	0.58–0.87	0.0009
Number of ER visits			0.1044			0.2156
1 vs. 0	1.06	1.00–1.13	0.0599	0.98	0.92–1.05	0.6037
2 vs. 0	1.08	0.97–1.20	0.1747	0.92	0.82–1.03	0.1503
≥3 vs. 0	1.08	0.96–1.23	0.2038	0.89	0.78–1.02	0.0855

Abbreviations: aHR = adjusted hazard ratio; COPD = chronic obstruction pulmonary disease; cHR = crude hazard ratio; ER = emergency room; USD = United States dollar.

**Table 4:** Subgroup analysis of dry eye disease for patients with and without rheumatoid arthritis.

	RA		Non-RA		Outcome Risk	
Subgroup	Incident Case	Incidence per 1000 Person-Years	Incident Case	Incidence per 1000 Person-Years	aHR (95% CI) ^†^	*p*
All patients	5594	23.14	2661	10.25	2.03 (1.93–2.13)	<0.0001
Age ≥ 65 years	1113	24.13	674	13.79	1.58 (1.43–1.75)	<0.0001
Age < 65 years	4481	22.91	1987	9.43	2.17 (2.06–2.30)	<0.0001
Male	1029	12.13	541	6.18	1.77 (1.59–1.97)	<0.0001
Female	4565	29.10	2120	12.32	2.10 (1.99–2.22)	<0.0001
CCI score = 0	4083	21.41	2163	9.76	2.03 (1.92–2.14)	<0.0001
CCI score = 1	1309	30.34	382	13.51	1.82 (1.62–2.06)	<0.0001
CCI score = 2	181	27.84	99	12.68	2.09 (1.62–2.70)	<0.0001
CCI score ≥ 3	21	15.76	17	8.66	2.18 (1.09–4.36)	0.0277
Use of systemic corticosteroids	2344	29.75	444	12.43	2.23 (2.01–2.47)	<0.0001
No use of systemic corticosteroids	3250	19.95	2217	9.90	1.95 (1.84–2.06)	<0.0001

Abbreviations: aHR = adjusted hazard ratio; CCI = Charlson comorbidity index; CI = confidence interval; RA = rheumatoid arthritis. ^†^ Adjusted for age (continuous), sex, insurance premium (categorical), coexisting diseases, Charlson comorbidity index score, use of systemic corticosteroids, number of hospitalizations, and number of emergency room visits.

**Table 5:** Univariate and multivariable analyses for corneal surface damage.

	Univariate			Multivariable		
	cHR	95% CI	*p*	aHR	95% CI	*p*
Rheumatoid arthritis	1.38	1.24–1.53	<0.0001	1.36	1.21–1.51	<0.0001
Age (years)	1.01	1.00–1.01	0.0003	1.01	1.00–1.01	0.0084
Sex, male vs. female	0.98	0.88–1.09	0.7022	0.97	0.87–1.08	0.5857
Insurance premium (USD/month)			0.0201			0.1126
501–800 vs. 0–500	1.09	0.97–1.23	0.1550	1.11	0.99–1.25	0.0796
≥801 vs. 0–500	0.89	0.78–1.02	0.0956	0.98	0.84–1.13	0.7310
Coexisting diseases						
Hypertension	1.12	0.98–1.27	0.0887	0.94	0.81–1.10	0.4247
Diabetes mellitus	1.23	1.03–1.46	0.0232	1.11	0.92–1.35	0.2828
Ischemic heart disease	1.20	1.00–1.45	0.0539	1.05	0.86–1.30	0.6265
COPD	1.20	0.98–1.48	0.0858	1.06	0.86–1.32	0.5762
Chronic liver disease	0.98	0.82–1.19	0.8669	0.90	0.74–1.09	0.2945
Chronic kidney disease	0.94	0.52–1.69	0.8271	0.77	0.42–1.40	0.3858
Cerebrovascular disease	1.37	1.08–1.74	0.0087	1.23	0.95–1.59	0.1143
Thyroid disease	1.17	0.80–1.71	0.4286	1.11	0.76–1.64	0.5849
Depressive disorder	0.96	0.54–1.69	0.8785	0.97	0.55–1.74	0.9277
Anxiety disorder	0.95	0.80–1.13	0.5850	0.88	0.73–1.05	0.1595
Sleeping disorder	0.89	0.74–1.07	0.1962	0.80	0.66–0.98	0.0282
Malignancy	1.26	0.94–1.69	0.1261	1.20	0.89–1.62	0.2427
Charlson Comorbidity Index			0.0061			0.2156
1 vs. 0	1.22	1.07–1.40	0.0040	1.05	0.90–1.21	0.5636
2 vs. 0	1.13	0.84–1.52	0.4393	1.05	0.77–1.42	0.7756
≥3 vs. 0	0.34	0.11–1.04	0.0585	0.32	0.10–0.98	0.0456
Use of systemic corticosteroids	1.24	1.10–1.39	0.0003	1.11	0.99–1.26	0.0824
Number of hospitalizations			0.2117			0.7818
1 vs. 0	1.19	1.00–1.42	0.0506	1.08	0.89–1.30	0.4333
2 vs. 0	1.12	0.78–1.61	0.5501	0.92	0.63–1.35	0.6698
≥3 vs. 0	1.18	0.77–1.82	0.4530	0.92	0.57–1.47	0.7211
Number of ER visits			0.0993			0.3919
1 vs. 0	1.07	0.92–1.25	0.3993	1.04	0.88–1.22	0.6767
2 vs. 0	1.29	1.00–1.65	0.0483	1.22	0.94–1.58	0.1279
≥3 vs. 0	1.25	0.94–1.67	0.1248	1.18	0.86–1.62	0.2991

Abbreviations: aHR = adjusted hazard ratio; COPD = chronic obstruction pulmonary disease; cHR = crude hazard ratio; ER = emergency room; USD = United States dollar.

## Data Availability

The data presented in this study are available on request from the corresponding author.

## Supplementary Materials

The following are available online at https://www.mdpi.com/article/10.3390/ijerph20021584/s1: Table S1. ICD-9-CM codes of exposure factors, coexisting diseases, and study outcomes; Figure S1. Flow diagram for patient selection.
