# Supplementary material for: Rheumatoid Arthritis Associated with Dry Eye Disease and Corneal Surface Damage: A Nationwide Matched Cohort Study
Source: Int J Environ Res Public Health. 2023 Jan 15;20(2):1584. doi: 10.3390/ijerph20021584 (PMC9861823; doi:10.3390/ijerph20021584)
Supplement: Supplementary file 1 [file ijerph-20-01584-s001.zip › Supplementary Table S1.pdf]

Supplementary Table S1. ICD-9-CM codes of exposure factors, coexisting diseases, and study outcomes

**Exposure factor**	
Rheumatic arthritis	725, 710.1, 710.3, 710.4, 714.0, 714.1, 714.2, 714.8
**Exclusion criteria**	
Dry eye disease	370.33, 372.53, 375.15, 710.2
Corneal ulcer	370.0
Recurrent corneal erosion	371.42
Corneal opacity	371
Interstitial and deep keratitis	370.5
Corneal neovascularization	370.6
Ocular burn	940
Open globe injury	871
**Coexisting disease**	
Hypertension	401-405
Diabetes mellitus	250
Ischemic heart disease	410-414
Chronic obstructive pulmonary disease	490, 491, 496
Chronic liver disease	571
Chronic kidney disease	585
Cerebrovascular disease	430-438
Thyroid disease	240-246
Depressive disorder	296.2, 296.3
Anxiety disorder	300
Sleeping disorder	307.41, 307.42, 327.0, 780.50, 780.52, 292.85
Malignancy	140-208, 230-234
**Study outcome**	
Dry eye disease	370.33, 372.53, 375.15, 710.2
Sjögren's syndrome	710.2
Corneal ulcer	370.0
Recurrent corneal erosion	371.42
Corneal opacity	371.0
