# Supplementary figures and images for: Rheumatoid Arthritis Associated with Dry Eye Disease and Corneal Surface Damage: A Nationwide Matched Cohort Study
Source: Int J Environ Res Public Health. 2023 Jan 15;20(2):1584. doi: 10.3390/ijerph20021584 (PMC9861823; doi:10.3390/ijerph20021584)

**Supplementary Figure S1.** Flow diagram for patient selection

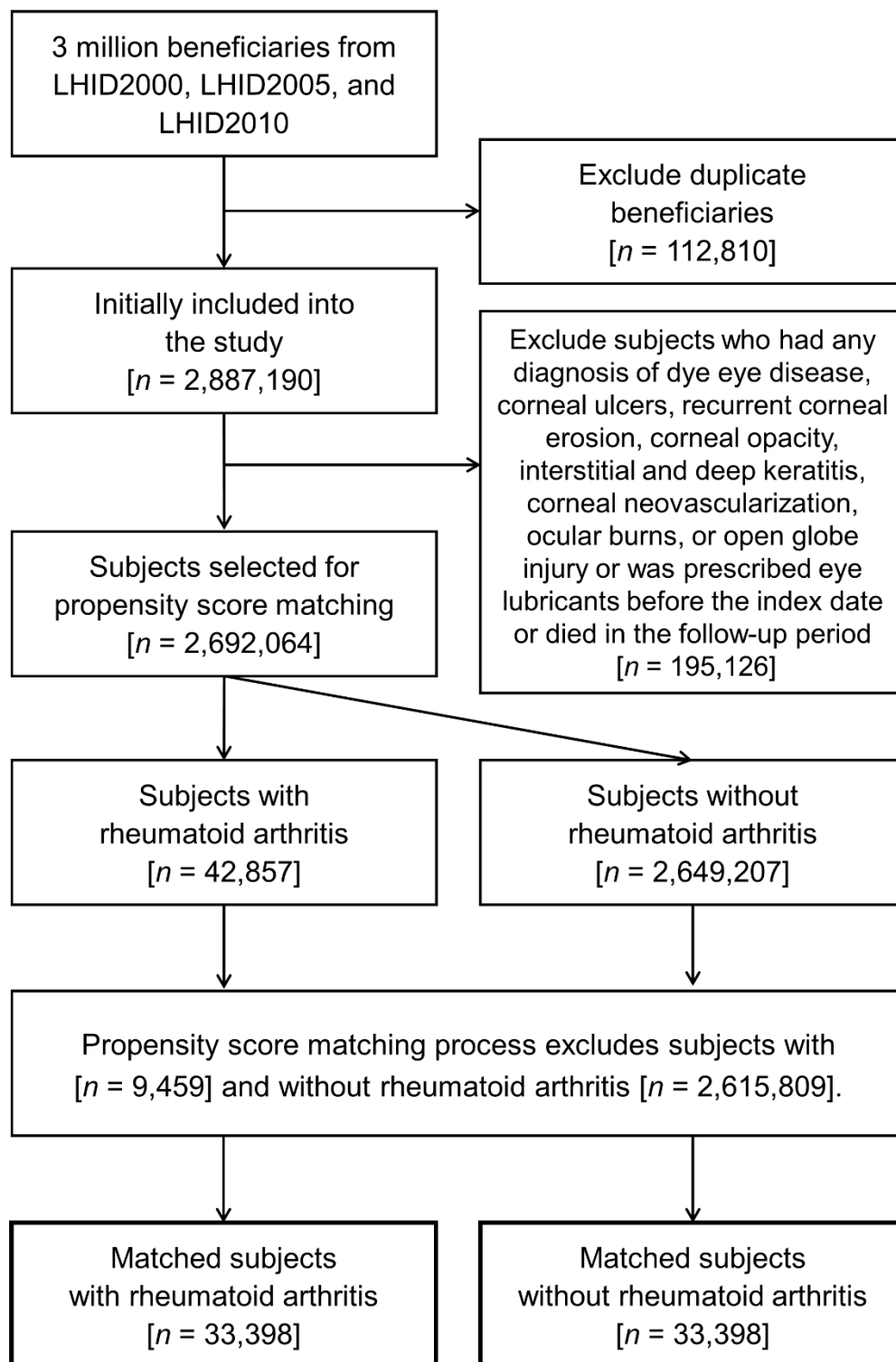

Supplement: Supplementary file 1 [file ijerph-20-01584-s001.zip › Supplementary Figure S1.pdf]
